# Revolutionizing Oncology Through AI: Addressing Cancer Disparities by Improving Screening, Treatment, and Survival Outcomes via Integration of Social Determinants of Health

**DOI:** 10.3390/cancers17172866

**Published:** 2025-08-31

**Authors:** Amit Kumar Srivastav, Aryan Singh, Shailesh Singh, Brian Rivers, James W. Lillard, Rajesh Singh

**Affiliations:** 1 Department of Microbiology, Biochemistry, and Immunology, Morehouse School of Medicine, Atlanta, GA 30310, USA; asrivastav@msm.edu (A.K.S.); shsingh@msm.edu (S.S.); jlillard@msm.edu (J.W.L.J.); 2 Hillgrove High School, Powder Springs, GA 30127, USA; aryansingh0319@gmail.com; 3 Cancer Health Equity Institute, Morehouse School of Medicine, Atlanta, GA 30310, USA; brivers@msm.edu

**Keywords:** artificial intelligence (AI), social determinants of health (SDOH), cancer, precision oncology, machine learning, explainable AI (XAI)

## Abstract

Background: Social determinants of health (SDOH) are critical contributors to cancer disparities, influencing prevention, early detection, treatment access, and survival outcomes. Addressing these disparities is essential in achieving equitable oncology care. Artificial intelligence (AI) is revolutionizing oncology by leveraging advanced computational methods to address SDOH-driven disparities through predictive analytics, data integration, and precision medicine. Methods: This review synthesizes findings from systematic reviews and original research on AI applications in cancer-focused SDOH research. Key methodologies include machine learning (ML), natural language processing (NLP), deep learning-based medical imaging, and explainable AI (XAI). Special emphasis is placed on AI’s ability to analyze large-scale oncology datasets, including electronic health records (EHRs), geographic information systems (GIS), and real-world clinical trial data, to enhance cancer risk stratification, optimize screening programs, and improve resource allocation. Results: AI has demonstrated significant advancements in cancer diagnostics, treatment planning, and survival prediction by integrating SDOH data. AI-driven radiomics and histopathology have enhanced early detection, particularly in underserved populations. Predictive modeling has improved personalized oncology care, enabling stratification based on socioeconomic and environmental factors. However, challenges remain, including AI bias in screening, trial underrepresentation, and treatment recommendation disparities. Conclusions: AI holds substantial potential to reduce cancer disparities by integrating SDOH into risk prediction, screening, and treatment personalization. Ethical deployment, bias mitigation, and robust regulatory frameworks are essential in ensuring fairness in AI-driven oncology. Integrating AI into precision oncology and public health strategies can bridge cancer care gaps, enhance early detection, and improve treatment outcomes for vulnerable populations.

## Simple Summary

Artificial intelligence (AI) has emerged as a transformative tool in oncology, offering innovative solutions to mitigate cancer disparities by integrating social determinants of health (SDOH) into predictive analytics, screening, treatment optimization, and prognostic modeling. This review explores AI-driven methodologies, including machine learning, deep learning, and natural language processing, to enhance early cancer detection and personalized treatment and improve patient outcomes, particularly in underserved populations. AI-powered medical imaging and histopathological analysis have improved the diagnostic accuracy, while predictive models facilitate precision oncology by tailoring therapies based on genetic, clinical, and socioeconomic factors. AI-driven geospatial analysis and telehealth solutions have also expanded cancer screening accessibility in resource-limited areas. Despite its potential, challenges such as algorithmic bias, the underrepresentation of minority groups in AI training datasets, and ethical concerns regarding data privacy remain. Addressing these barriers through fairness-aware AI models, federated learning, and regulatory oversight is critical in achieving equitable cancer care.

## 1. Introduction

**Figure 1:**
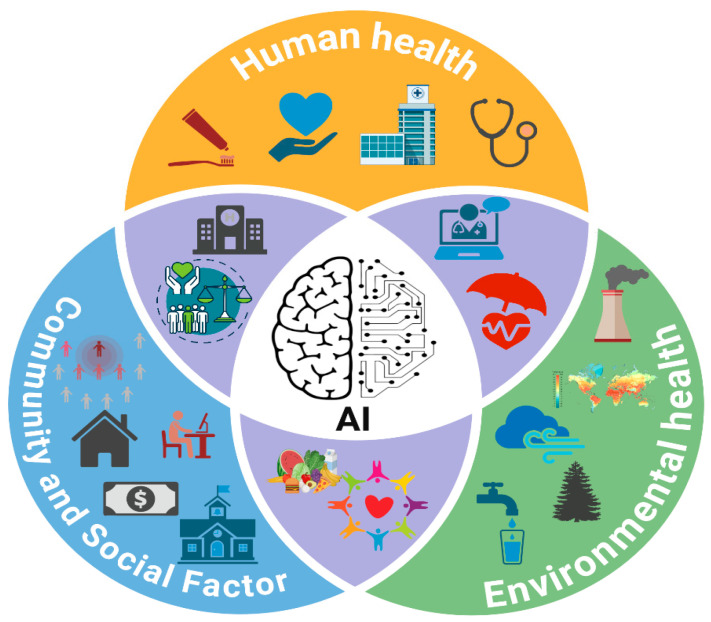
AI integration with SDOH factors (human health, environmental health, and social factors) to address cancer health disparities.

Cancer is a complex and multifaceted disease influenced by a combination of genetic, environmental, and social factors, making its prevention, diagnosis, and treatment a significant global challenge. Despite advancements in precision medicine, early detection, and targeted therapies, disparities in cancer incidence, treatment accessibility, and survival rates persist across different populations [1]. Biological determinants do not solely drive these disparities but are deeply rooted in systemic inequities within healthcare infrastructure, socioeconomic status, environmental exposures, and social determinants of health (SDOH). Social determinants of health (SDOH), including economic stability, education, healthcare accessibility, and geographic location, play a pivotal role in shaping cancer-related outcomes (Figure 1) [2]. Marginalized and underserved communities often face financial, geographic, and institutional barriers that delay cancer detection and limit access to optimal treatment strategies. Individuals from lower socioeconomic backgrounds, racial and ethnic minorities, and rural populations experience higher cancer mortality rates due to reduced access to screening programs, late-stage diagnoses, and disparities in treatment adherence [3].

Artificial intelligence (AI) has emerged as a transformative tool in oncology, offering novel approaches to mitigating these disparities through predictive analytics, big data integration, and precision medicine strategies. AI-powered models, including machine learning (ML), deep learning (DL), and natural language processing (NLP), have shown promising results in enhancing early cancer detection, optimizing personalized treatment plans, and improving prognostic predictions. Integrating AI with large-scale datasets, such as electronic health records (EHRs), genomic profiles, imaging modalities, and geospatial data, enables a more comprehensive understanding of cancer disparities and facilitates targeted interventions [4,5].

This review explores the intersection of AI and SDOH in addressing cancer inequities. It examines how AI-driven technologies enhance cancer screening, improve treatment accessibility, and refine predictive modeling for personalized oncology care. Additionally, it highlights challenges related to AI biases, ethical considerations, data privacy, and the regulatory landscape governing AI implementation in oncology. By integrating AI-driven innovations into public health strategies and precision medicine frameworks, this review aims to provide insights into how AI can bridge the existing gaps in cancer care and contribute to equitable healthcare outcomes.

## 2. Methodology

**Figure 2:**
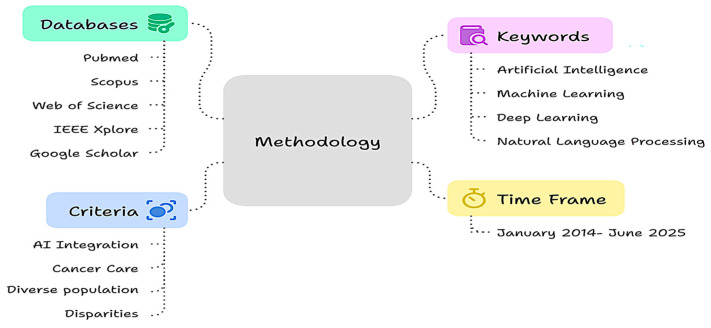
Research framework methodology to address cancer disparities through AI integration.

A structured literature search was conducted to ensure the comprehensive coverage of relevant studies and developments in the field (Figure 2). The search strategy incorporated multiple reputable biomedical and interdisciplinary databases, including PubMed, Scopus, Web of Science, IEEE Xplore, and Google Scholar. A search was conducted to capture the research on artificial intelligence (AI), social determinants of health (SDOH), and cancer disparities. The core search terms included combinations of keywords such as “artificial intelligence”, “machine learning”, “deep learning”, “natural language processing”, “explainable AI”, “social determinants of health”, “cancer disparities”, “oncology precision medicine”, “health equity”, and “clinical trial diversity”. The literature search covered peer-reviewed articles published between January 2014 and June 2025, focusing on studies reporting on AI methodologies and applications in oncology that explicitly address or incorporate SDOH-related variables. Emphasis was placed on high-impact journals in oncology, public health, digital medicine, and biomedical informatics. Articles were selected based on their methodological rigor, relevance to AI integration in cancer care, representation of diverse populations, and contributions to addressing disparities. The reference lists of key articles and systematic reviews were manually screened to identify additional pertinent publications.

The literature selection process was iterative and thematic, ensuring the balanced inclusion of foundational concepts, current applications, and emerging directions in AI-driven oncology disparity research. This approach enhanced the transparency while preserving the narrative scope required to synthesize multidisciplinary perspectives in this evolving field.

## 3. The Role of Social Determinants of Health (SDOH) in Cancer Disparities

### 3.1. Socioeconomic, Healthcare, and Environmental Determinants of Cancer Disparities

Cancer remains one of the most significant global health burdens, yet the outcomes of cancer diagnosis and treatment are not uniform across all populations. In their cohort study, Pinheiro, L.C. et al. reported that, while genetic predisposition and lifestyle choices are major contributors to cancer risk, social, economic, and environmental factors, collectively known as social determinants of health (SDOH), play a fundamental role in shaping cancer incidence, early detection, treatment accessibility, and survival rates (Table 1) [6]. These non-medical factors create structural barriers that disproportionately affect marginalized and underserved populations, leading to disparities in healthcare access and outcomes [6].

Socioeconomic status (SES) is a critical determinant of health, influencing an individual’s ability to access preventive screenings, timely diagnosis, and appropriate treatment. Individuals from low-income communities often face financial barriers that prevent them from undergoing routine cancer screenings, receiving early diagnoses, and adhering to prescribed treatments. In 2022, Fnu, N. et al. reported that the costs of cancer therapies, hospital visits, and post-treatment care can be prohibitive, leading to delayed intervention and increased mortality rates [7]. Timely cancer detection is essential in improving survival rates, yet access to high-quality oncology care remains unevenly distributed [7]. Many rural and underserved urban communities lack specialized cancer centers, trained oncologists, and state-of-the-art diagnostic technologies, making early detection challenging. This geographic disparity in healthcare infrastructure significantly delays diagnosis and treatment initiation, leading to higher incidences of late-stage cancers among historically marginalized populations [8].

In the United States, Zavala, V.A. et al. found that specific ethnic groups, such as African American, European American, or Hispanic populations, were disproportionately diagnosed with advanced-stage breast, colorectal, and lung cancer, largely due to reduced access to screening programs such as mammograms, colonoscopies, and low-dose CT scans for lung cancer [9]. The lack of routine screenings in these populations directly correlates with higher mortality rates, demonstrating the urgent need for targeted interventions to increase screening accessibility [10]. Furthermore, in their study, Larsen, K. et al. reported that environmental factors also play a crucial role in cancer disparities, as exposure to industrial pollutants, carcinogens, and inadequate living conditions can elevate cancer risk. Communities situated near industrial zones, landfills, or high-traffic areas are often exposed to higher levels of air pollution, heavy metals, and chemical toxins, increasing their likelihood of developing cancers such as lung, bladder, and liver cancer [11].

### 3.2. AI’s Potential in Addressing SDOH-Driven Cancer Inequities

**Figure 3:**
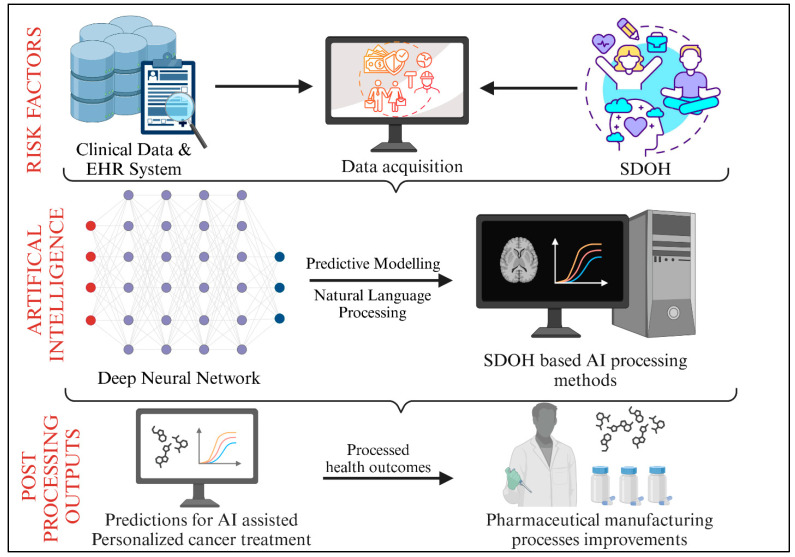
Workflow demonstrating AI integration in healthcare, from clinical data acquisition via EHR systems to SDOH-based predictive modeling and NLP, resulting in personalized cancer treatment predictions and pharmaceutical process optimization.

Artificial intelligence (AI) has emerged as a transformative tool in healthcare, providing data-driven solutions to mitigate disparities in cancer care. AI can process large-scale, multidimensional datasets, allowing researchers and clinicians to identify cancer risk factors, predict disease progression, and develop tailored treatment plans based on individual genetic, clinical, and socioeconomic characteristics. AI technologies such as machine learning (ML), deep learning (DL), natural language processing (NLP), and predictive analytics can enhance early cancer detection, optimize treatment decisions, and bridge healthcare gaps for underserved populations (Figure 3) [12].

Tiwari, A. et al. reported that AI-powered screening tools have revolutionized cancer diagnostics by integrating advanced computational techniques such as computer vision, radiomics, and deep learning models to enhance the detection accuracy and efficiency [13]. These AI-based imaging solutions analyze mammograms, computed tomography (CT) scans, magnetic resonance imaging (MRI), and histopathological slides with high precision, allowing for the earlier and more accurate identification of malignant lesions [13]. In a recent study in 2025, Yao, I.Z. et al. showed that, by leveraging deep learning algorithms, AI models can detect subtle tumor markers, microcalcifications, and abnormal tissue patterns that might be imperceptible to human radiologists, significantly reducing false-negative rates and improving early cancer diagnosis [14].

In 2020, Chan, H.P. et al. reported that AI-driven computer-aided detection (CAD) systems have demonstrated remarkable capabilities in identifying abnormalities across various imaging modalities [15]. In mammography, AI models assist in distinguishing between benign and malignant breast lesions, improving the diagnostic accuracy and supporting radiologists in detecting early-stage breast cancer [15]. In one study, Cellina, M. et al. showed that, in lung cancer screening, AI-powered low-dose CT (LDCT) analysis enhances the detection of pulmonary nodules, reducing unnecessary biopsies while ensuring timely intervention for high-risk patients [16]. Similarly, in 2025, Makar, J. et al. reported that, in colorectal cancer, AI-assisted colonoscopy systems equipped with real-time polyp detection algorithms significantly improved the adenoma detection rate, leading to better patient outcomes [17]. Meanwhile, Wang, J. et al.’s study outlined that histopathological analysis has also benefited from AI-driven automation, where deep learning-based whole-slide imaging (WSI) models assist pathologists in identifying cancerous cells with greater speed and consistency [18]. Moreover, Rituraj et al. mentioned that precision oncology has revolutionized cancer treatment by tailoring therapies based on an individual’s genetic profile, tumor characteristics, and SDOH factors. AI-driven multi-omics analysis enables oncologists to identify genetic mutations, drug response biomarkers, and immune system interactions, allowing personalized treatment regimens [19]. In their study, Rafique, R. et al. indicated that machine learning models can also predict chemotherapy resistance, immunotherapy responses, and radiotherapy effectiveness based on biological and social health determinants [20]. For example, the AI-driven stratification of African American vs. European American colorectal cancer patients revealed key differences in tumor biology, drug metabolism, and immune response, influencing treatment efficacy. Integrating these insights into personalized cancer care plans can help to reduce disparities in treatment outcomes [20].

### 3.3. AI as a Tool to Reduce Cancer Disparities

Artificial intelligence (AI) has emerged as a transformative tool in oncology, offering innovative solutions to mitigate cancer disparities by leveraging predictive analytics, geospatial modeling, and community health interventions. Traditional cancer care models often fail to account for systemic inequities in healthcare access, socioeconomic barriers, and geographic limitations, which contribute to disparities in cancer screening, treatment, and survival outcomes. By integrating AI-driven insights into public health strategies and clinical policies, healthcare organizations can enhance screening accessibility, optimize resource allocation, and improve participation in clinical trials for underrepresented populations.

In their study, Lee, D.C. et al. observed that cancer screening remains one of the most effective strategies for reducing cancer-related morbidity and mortality, yet inequitable access to early detection programs persists, particularly in rural, low-income, and racial minority communities [21]. Many underserved regions lack adequate healthcare infrastructure, trained oncologists, and diagnostic facilities, leading to delays in cancer detection and a higher prevalence of late-stage diagnoses [21]. AI-powered public health interventions aim to bridge these gaps using geospatial analysis and predictive modeling to optimize cancer screening accessibility and resource allocation [22].

**Figure 4:**
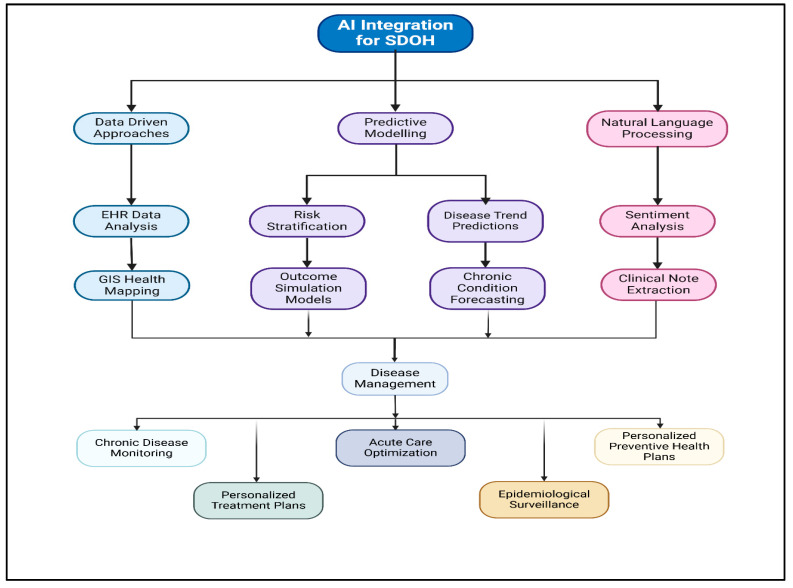
AI integration framework for SDOH, demonstrating data-driven approaches, predictive modeling, and NLP for disease management and health outcomes.

Geospatial analysis, combined with AI-driven geographic information systems (GIS), machine learning algorithms, and real-time cancer registry data, enables public health officials to pinpoint geographic disparities in the cancer burden. In a recent work, Zhang, B. et al. showed that AI models can assess demographic risk factors, healthcare facility distributions, and population-level cancer incidence rates to identify regions with disproportionately high cancer mortality and low screening uptake [23]. For example, deep learning models trained on historical cancer screening data can predict which zip codes, counties, or urban districts are at a higher risk for undiagnosed cancer cases due to insufficient medical infrastructure, financial constraints, or cultural barriers to healthcare-seeking behavior [23]. These AI-powered insights allow policymakers to implement targeted interventions, such as mobile cancer screening units, community outreach programs, and digital health initiatives, in areas with the greatest need [24]. Furthermore, the flowchart below (Figure 4) illustrates how AI leverages SDOH through data analysis, predictive modeling, and NLP to improve disease management, personalized care, and public health strategies.

### 3.4. AI for Equitable Cancer Research and Clinical Trial Representation

Equitable representation in clinical research is critical in ensuring that novel cancer therapies are effective across diverse populations. However, racial and ethnic minorities, low-income patients, and individuals from rural communities remain underrepresented in oncology clinical trials, limiting the generalizability of research findings and the efficacy of precision medicine interventions. AI has the potential to transform clinical trial recruitment by identifying eligible patients from diverse backgrounds, improving accessibility, and reducing systemic barriers to participation [25].

Traditional clinical trial recruitment relies on physician referrals, the manual screening of patient records, and self-enrollment processes, which often result in low participation rates among minority and socioeconomically disadvantaged patients. AI-driven recruitment platforms automate and streamline patient identification by using natural language processing (NLP) algorithms and machine learning models to scan electronic health records (EHRs), cancer registries, and genomic databases for patients who meet trial eligibility criteria [26]. Ligero, M. et al. demonstrated in their studies that AI-powered NLP models can extract key clinical and demographic attributes from unstructured EHR data, such as tumor types, biomarker statuses, treatment histories, and socioeconomic indicators, to generate personalized recommendations for clinical trial enrollment [27]. These AI-assisted screening tools reduce the burden on oncologists and research coordinators, increasing the efficiency and inclusivity of trial recruitment efforts [27].

In their recent work, Sedano, R. et al. showed that AI can also help to mitigate barriers to clinical trial participation by addressing logistical, financial, and informational challenges disproportionately affecting underrepresented populations [28]. AI-driven chatbots and virtual assistants provide patients with culturally tailored education about clinical trials, addressing common concerns and misconceptions about participation. These digital tools help to increase awareness and trust in medical research among communities historically excluded from clinical studies [28]. Additionally, Yelne, S. et al. described that AI-powered predictive modeling tools can assess financial barriers, transportation limitations, and social determinants of health to identify patients who may require additional support to participate in clinical research. By integrating SDOH-driven AI insights into trial recruitment strategies, research institutions can offer targeted interventions such as transportation assistance, financial reimbursements, and language translation services, ensuring that participation is equitable and accessible for all patient populations [29].

## 4. AI Applications in Cancer Care and Disparities

### 4.1. AI-Driven Advances in Cancer Diagnostics and Screening

Early cancer detection is critical in improving patient outcomes and reducing mortality rates. AI can expand cancer screening accessibility in low-resource settings by reducing the reliance on highly specialized radiologists and oncologists (Table 2). Goel, I. et al. reported that AI-powered mobile screening units equipped with cloud-based AI models can facilitate early breast and cervical cancer detection in rural areas, where access to specialized oncology services is limited [30]. AI-based telepathology platforms enable the remote analysis of biopsy samples, allowing for the efficient triaging of high-risk cases and timely interventions [30]. Additionally, the studies performed by Broggi, G. et al. reported that AI-driven natural language processing (NLP) can analyze electronic health records (EHRs) and demographic data to identify populations at a higher risk for late-stage cancer diagnosis, prompting targeted outreach programs [31].

### 4.2. AI in Personalized Cancer Therapy and Treatment Optimization

Personalized cancer treatment integrates molecular, clinical, and social determinant of health (SDOH) data to optimize therapy selection. AI models leveraging multi-omics data (genomics, proteomics, and transcriptomics) could predict tumor responsiveness to chemotherapy, immunotherapy, and targeted drugs with higher precision. Banumathi, K. et al. reported in their studies that reinforcement learning (RL)-based AI systems have been developed to suggest adaptive treatment regimens that are dynamically adjusted based on tumor progression, patient responses, and socioeconomic factors [32]. These AI frameworks analyze real-world evidence from clinical trials, patient registries, and population-level health disparities to personalize therapy recommendations [32]. Many AI-driven predictive models have shown promise in forecasting chemotherapy toxicity, patient responses to immunotherapy, and radiation sensitivity (Table 3). Machine learning models incorporating patient comorbidities, treatment history, genetic biomarkers, and SDOH variables can predict the likelihood of adverse reactions to chemotherapy agents. For radiotherapy planning, Fu, Y. et al. showed that AI-based dose optimization algorithms analyze tumor radiosensitivity to customize treatment regimens while minimizing radiation exposure to healthy tissues [33]. AI-driven adaptive radiotherapy systems continuously adjust treatment parameters based on real-time tumor volume changes, enhancing precision in radiation oncology [33].

### 4.3. AI for Cancer Prognosis and Survival Prediction

Machine learning (ML) and deep learning (DL) advancements have significantly improved cancer prognosis by enabling accurate predictions of tumor recurrence, metastasis, and survival outcomes. Supervised and unsupervised ML models, including gradient boosting algorithms, deep neural networks (DNNs), and ensemble learning techniques, have been used to analyze longitudinal patient data, clinical biomarkers, genomic alterations, and treatment responses to generate individualized risk assessments. These models facilitate the quantitative evaluation of recurrence probabilities, optimize survival predictions under different therapeutic regimens, and assess the influence of social determinants of health (SDOH) on long-term outcomes [34].

Integrating these AI-driven predictive models into oncology decision support systems (DSS) allows oncologists to stratify patients into high-risk and low-risk cohorts, guiding personalized follow-up strategies, early intervention planning, and adaptive post-treatment monitoring. In a similar study, Russo, V. et al. indicated that, by leveraging real-world evidence from electronic health records (EHRs), imaging datasets, and multi-omics profiles, AI models enhance precision oncology, ensuring proactive and data-driven clinical decision making to improve patient outcomes and overall cancer care efficiency [35].

### 4.4. AI-Powered Real-Time Monitoring for Treatment Adherence in Low-Income Patients

Treatment adherence remains a significant challenge, particularly in low-income and underserved populations, where patients often face financial barriers, transportation limitations, and social support deficiencies. AI-powered remote monitoring systems, incorporating wearable devices, smartphone applications, and telehealth platforms, enable the real-time tracking of treatment adherence. Cunha Reis, T. reported that AI-based natural language processing (NLP) tools analyze patient-reported symptoms and healthcare utilization patterns to identify those at risk of non-adherence or treatment discontinuation [36]. These insights facilitate early interventions, personalized support programs, and tele-oncology follow-ups, improving overall treatment outcomes [36].

### 4.5. AI-Based Multi-Omics Integration for Personalized Prognostics

Integrating multi-omics data with artificial intelligence (AI) has revolutionized personalized prognostics in oncology, enabling data-driven risk assessment, treatment optimization, and real-time clinical decision making. A recent study performed by Verlingue, L. et al. showed that AI-driven deep reinforcement learning (DRL) and machine learning (ML) models can analyze electronic health records (EHRs), genomic profiles, transcriptomic signatures, and proteomic biomarkers to predict tumor progression, therapy responses, and long-term survival outcomes [37].

AI-enhanced multi-omics integration strengthens the predictive power of oncology models, enabling the more precise stratification of patients based on tumor heterogeneity, genetic predispositions, and environmental influences. The synergy between AI, predictive analytics, and real-time patient monitoring supports early cancer detection, individualized treatment selection, and improved survival rates. However, addressing key challenges such as algorithmic bias, limited diversity in training datasets, and disparities in AI accessibility is crucial in ensuring equitable AI-driven oncology care. Future advancements should focus on developing bias-free, interpretable AI frameworks incorporating social determinants of health (SDOH), ensuring that precision oncology strategies benefit all populations regardless of socioeconomic status, race, or geographic location [38].

## 5. Addressing Challenges in AI-Driven Cancer Care

### 5.1. Underrepresentation of Minority Groups in AI Training Datasets

AI models rely on large-scale datasets to train deep learning algorithms, improve pattern recognition, and generate accurate predictions. However, a major limitation in oncology-focused AI systems is the underrepresentation of racial and ethnic minority groups in these training datasets. Most publicly available cancer imaging, genomics, and clinical datasets are skewed towards populations of European ancestry, leading to disparities in AI models’ generalizability and accuracy [39]. AI models trained predominantly on breast cancer imaging datasets from Caucasian women have demonstrated higher sensitivity and specificity for tumor detection in White patients compared to African American and Hispanic patients. This results in misclassification, increased false-negative rates, and delayed diagnoses for minority populations, exacerbating existing healthcare disparities. In lung cancer detection, AI models trained on non-diverse imaging datasets have shown lower performance in detecting lung nodules in Asian and African American patients [40].

### 5.2. AI Limitations in Capturing Non-Clinical SDOH Factors Affecting Cancer Care

While AI has shown remarkable progress in cancer diagnostics and treatment planning, it remains limited in capturing the full scope of non-clinical SDOH factors that influence patient outcomes. Traditional AI models primarily rely on biological markers, imaging data, and clinical histories, often neglecting socioeconomic barriers, environmental exposures, healthcare accessibility, and psychosocial determinants, which significantly impact cancer progression and survival rates [41]. Furthermore, AI-driven predictive models for chemotherapy adherence often focus on molecular and pharmacogenomic data and fail to integrate real-world factors such as transportation challenges, financial toxicity, caregiver availability, and language barriers. Similarly, cancer recurrence prediction models trained only on clinical variables overlook stress levels, nutrition, housing conditions, and access to follow-up care, all of which play a pivotal role in patient outcomes [4].

### 5.3. Strategies for Bias Mitigation, Model Fairness Testing, and Federated Learning

Fairness, transparency, and impartiality in AI-driven oncology require robust bias mitigation strategies, fairness-aware AI models, and decentralized learning approaches. Bias auditing is a critical step in AI model validation, involving continuous fairness assessments to detect performance disparities across racial, ethnic, and socioeconomic subgroups. These audits facilitate the recalibration of prediction thresholds for high-risk populations, ensuring that AI-based cancer screening and diagnostic tools maintain clinical reliability across diverse patient cohorts [42]. A pivotal innovation in equitable AI development is federated learning. This decentralized approach enables AI models to be trained across multiple institutions, geographic regions, and demographic groups without directly sharing sensitive patient data. Unlike traditional centralized training methods, federated learning preserves data privacy and security while ensuring model robustness across heterogeneous populations. This technique is particularly effective in mitigating racial and socioeconomic biases in AI-driven cancer screening, diagnosis, and treatment planning by fostering cross-institutional knowledge integration and ethical AI deployment in precision oncology [43].

### 5.4. Challenges in Clinical Trials and Oncology Decision Making

One of the major challenges in AI-driven oncology is the underrepresentation of racial and ethnic minorities in clinical trials, which has profound implications for cancer treatment personalization and drug efficacy assessment. Traditional clinical trials have historically excluded or under-enrolled African American, Hispanic, and Indigenous populations, leading to limited data on treatment responses and adverse effects for these groups [42]. When AI models are trained on clinical trial data that lack diversity, their ability to predict drug efficacy, toxicity risks, and survival probabilities in minority patients is significantly compromised. For instance, AI-driven precision medicine algorithms trained on genomic data predominantly from individuals of European ancestry may not accurately predict targeted therapy responses in African and Asian populations, leading to suboptimal treatment recommendations [44].

## 6. AI-Enabled Interventions to Reduce Cancer Disparities

### 6.1. AI for Public Health and Cancer Screening Accessibility

Geospatial analysis and AI-powered geographic information systems (GIS) have revolutionized public health surveillance and cancer screening accessibility. AI-driven GIS models analyze demographic, environmental, and healthcare infrastructure data to identify cancer hotspots—regions with higher-than-expected cancer incidence and mortality rates [45]. AI-based spatial epidemiology models integrate real-time cancer registry data, electronic health records (EHRs), and environmental exposure metrics to determine regions with low cancer screening participation and high numbers of late-stage cancer diagnoses. For example, in Wang, L.’s study, the utilization of deep neural networks (DNNs) and reinforcement learning algorithms has demonstrated how AI can predict areas with insufficient mammography and colorectal cancer screening coverage [46]. These models factor in transportation barriers, healthcare provider density, and socioeconomic indicators to pinpoint at-risk populations that require targeted screening interventions [46].

### 6.2. AI-Driven Policy Recommendations for Equitable Oncology Care

AI-powered healthcare policy modeling enables evidence-based funding allocation and cancer control program design. Machine learning algorithms analyze epidemiological trends, healthcare costs, and SDOH data to predict the future cancer burden and recommend targeted policy interventions. Predictive modeling tools integrate real-world cancer incidence rates with healthcare utilization metrics to help policymakers to allocate resources to high-need areas. AI-driven simulations provide insights into the long-term impacts of different cancer prevention strategies, such as screening expansions, genetic testing availability, and tobacco control policies [47]. AI-based policy recommendation systems also play a vital role in addressing racial and socioeconomic disparities in cancer outcomes. AI-driven impartial audits assess whether healthcare policies disproportionately benefit specific groups while neglecting underserved populations. By analyzing historical disparities in cancer care delivery, AI helps policymakers to implement bias-free, inclusive cancer care policies [47].

### 6.3. AI-Driven Strategies to Improve Access to Clinical Trials and Precision Medicine

Clinical trials are the foundation of oncology research and precision medicine, yet the underrepresentation of racial and ethnic minorities remains a persistent challenge. AI transforms clinical trial recruitment by identifying eligible participants from diverse demographic backgrounds, ensuring greater inclusivity in cancer research. Smiley, A. et al. showed that natural language processing (NLP) algorithms could analyze unstructured clinical notes, genomic databases, and patient registries to match underrepresented populations with relevant cancer trials [48]. AI-driven real-time patient recruitment platforms streamline eligibility screening and trial enrollment, expanding access for low-income and rural patients who may otherwise be excluded from cutting-edge cancer treatments [48]. Machine learning models also predict barriers to trial participation, such as transportation difficulties, language barriers, and financial constraints. AI-based solutions address these challenges by integrating telemedicine consultations, remote monitoring technologies, and financial assistance programs into clinical trial designs [49].

## 7. Ethical Considerations in AI-Driven Cancer Care

### 7.1. Ensuring Patient Data Protection and Regulatory Compliance

AI-driven cancer models rely on large-scale datasets, including electronic health records (EHRs), genomic sequences, imaging scans, and patient-reported outcomes, to train and optimize predictive algorithms. However, the uncertainty regarding data security and the utilization of sensitive personal health information (PHI) in AI models raise serious concerns about data privacy, security, and ethical handling (Table 4). Furthermore, medical data breaches can have severe consequences, including identity theft, unauthorized access, discrimination, and loss of patient trust in AI-driven healthcare innovations [50]. The Health Insurance Portability and Accountability Act (HIPAA) in the United States and the General Data Protection Regulation (GDPR) in Europe set strict guidelines aimed at protecting patient data, ensuring consent, and limiting data sharing. However, AI-driven oncology applications require continuous compliance with these regulatory frameworks to mitigate potential ethical risks (Table 5) [51].

### 7.2. AI-Based Privacy-Preserving Techniques and Federated Learning

The integration of AI in oncology necessitates stringent privacy-preserving techniques to ensure the security of sensitive patient data while maintaining model performance across diverse populations. Traditional machine learning models rely on centralized data aggregation, which poses risks related to data breaches, unauthorized access, and ethical concerns regarding patient confidentiality [53]. To address these challenges, privacy-preserving AI techniques, such as federated learning, homomorphic encryption, and differential privacy, have emerged as robust solutions in AI-driven cancer research. Federated learning (FL) is a decentralized AI training approach that enables multiple institutions to collaboratively develop predictive oncology models without sharing raw patient data. Instead of transferring patient records to a central repository, FL allows AI models to be trained locally on institution-specific datasets, with only encrypted model updates being shared across participating sites. This approach preserves data confidentiality and facilitates AI model generalizability by incorporating multi-institutional and geographically diverse datasets, reducing bias and enhancing the predictive accuracy for underrepresented populations [54]. Complementary to FL, homomorphic encryption enables AI models to perform computations on encrypted data, ensuring that sensitive patient information remains secure throughout the model training process. Differential privacy further enhances security by injecting controlled statistical noise into datasets, preventing the re-identification of individual patient records while still allowing meaningful insights to be extracted. These privacy-preserving AI techniques are essential for regulatory compliance with frameworks such as HIPAA (Health Insurance Portability and Accountability Act) and GDPR (General Data Protection Regulation), ensuring ethical AI deployment in oncology. By implementing federated learning and advanced cryptographic methods, AI-driven cancer research can maximize the predictive power while upholding data integrity, security, and patient trust in precision medicine initiatives [55].

### 7.3. Explainable AI (XAI) to Enhance Transparency in Cancer Decision Making

**Figure 5:**
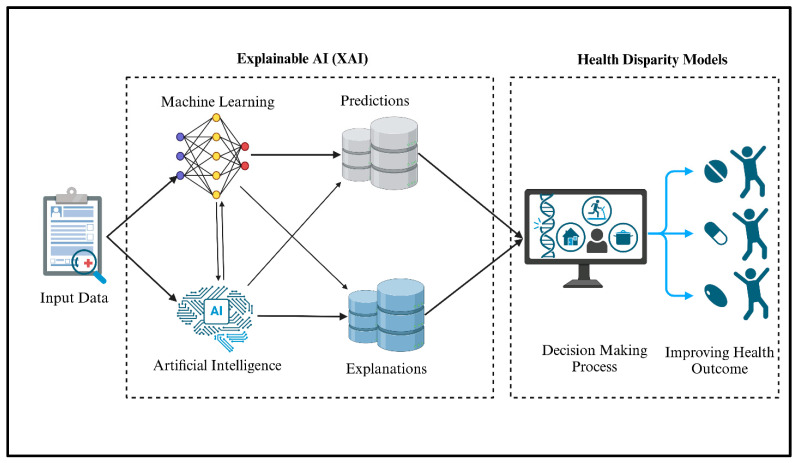
Explainable AI (XAI) framework for health disparity models, integrating machine learning predictions and explanations to improve decision making and health outcomes for cancer.

As artificial intelligence (AI) becomes increasingly integrated into oncology, ensuring transparency and interpretability in AI-driven clinical decisions is paramount. Traditional deep learning models—particularly black-box algorithms like deep neural networks (DNNs) and convolutional neural networks (CNNs)—often lack explainability, making it difficult for oncologists to trust and validate AI-generated predictions. Explainable AI (XAI) aims to address this limitation by providing insights into how AI models arrive at specific conclusions, enhancing the interpretability, physician trust, and regulatory compliance in cancer diagnostics and treatment planning (Figure 5) [56].

XAI techniques, such as Shapley Additive Explanations (SHAP), Local Interpretable Model-Agnostic Explanations (LIME), and attention mechanisms, enable AI systems to highlight critical features influencing model predictions, such as tumor morphologies, genomic alterations, or patient demographics. In terms of medical imaging, in Ayaz, H. et al.’s work, saliency maps and gradient-weighted class activation mapping (Grad-CAM) were used to visualize regions of interest in radiology scans, ensuring that AI models focused on clinically relevant tumor features [57]. These explainability tools empower oncologists to validate AI-driven diagnoses, reduce misclassification risks, and improve patient–physician communication by offering interpretable risk assessments rather than opaque outputs [57].

Moreover, XAI is crucial for bias detection and mitigation in AI-driven oncology. By revealing model decision pathways, XAI can help to identify disparities in cancer risk predictions across different demographic groups, ensuring that AI systems do not perpetuate existing healthcare inequalities. Regulatory bodies such as the Food and Drug Administration (FDA) and European Medicines Agency (EMA) emphasize the need for explainability in AI-based medical devices, reinforcing XAI’s role in ethical and accountable AI deployment in oncology. As AI continues to shape precision medicine, integrating XAI frameworks will ensure fairness, trust, and the clinical adoption of AI-driven cancer care solutions [58].

Ethical considerations in AI-driven oncology require a multifaceted approach encompassing data privacy protection, regulatory compliance, model interpretability, and bias mitigation strategies. By implementing HIPAA/GDPR-compliant AI frameworks, explainable AI (XAI) methodologies, and continuous fairness testing, AI can contribute to more equitable, transparent, and trustworthy cancer care. Future advancements must focus on integrating SDOH-driven insights, enhancing physician–AI collaboration, and developing global AI ethics standards to ensure that AI benefits all cancer patients—regardless of socioeconomic status, race, or geographic location [59].

## 8. Future Directions of AI in Cancer Disparity Research

### 8.1. Advances in AI-Driven Precision Oncology

Integrating artificial intelligence (AI) in oncology has demonstrated significant potential in enhancing cancer diagnostics, treatment planning, and equitable healthcare delivery. However, the future of AI in cancer disparities research will be driven by advancements in precision oncology, multi-omics integration, and the incorporation of social determinants of health (SDOH) into AI-driven decision-making frameworks. The next generation of AI-powered oncology models will emphasize longitudinal survival predictions, explainable AI (XAI), federated learning, and AI-driven community health interventions. Additionally, the emergence of environmental and lifestyle-based AI prevention strategies will allow for proactive rather than reactive cancer care, thereby improving population-wide cancer outcomes while addressing healthcare inequities [60].

Precision oncology has undergone rapid evolution with the advent of multi-omics technologies, which integrate genomic, transcriptomic, proteomic, epigenomic, and metabolomic data to develop personalized treatment strategies [61]. AI-driven multi-omics integration enables comprehensive cancer profiling, allowing oncologists to tailor therapeutic interventions based on an individual’s molecular signature, rather applying than a one-size-fits-all approach. Deep learning algorithms, particularly Transformer-based neural networks and graph-based AI models, can process complex multi-omics datasets to identify cancer-specific biomarkers, drug resistance mechanisms, and tumor microenvironment interactions. AI-enhanced multi-modal learning models are also being developed to synthesize heterogeneous datasets (e.g., genetic mutations, histopathological features, imaging data, and clinical history) to predict optimal treatment regimens for individual patients [5,62].

Integrating AI into multi-omics oncology research has yielded promising results in identifying novel drug targets and optimizing immunotherapy selection. For instance, the work performed by Yang, B. et al. showed that AI-powered deep neural networks can be used to predict the tumor mutational burden (TMB) and microsatellite instability (MSI) status, which are key indicators of a patient’s response to immune checkpoint inhibitors (ICIs) [63]. Future advancements in federated multi-omics learning frameworks will further reduce algorithmic bias and improve AI models’ generalizability across diverse populations [63].

### 8.2. AI Models Predicting Long-Term Cancer Survival and Quality of Life

One of the most promising frontiers in AI-driven oncology is the development of longitudinal survival prediction models that assess cancer recurrence risks, metastasis probabilities, and post-treatment quality of life (QoL). Traditional survival prediction models rely on Kaplan–Meier curves and Cox proportional hazard regression, which have limitations in handling high-dimensional, time-dependent clinical data. AI-driven recurrent neural networks (RNNs) and Transformer-based time-series models overcome these limitations by incorporating dynamic patient data over time, enabling real-time risk stratification and personalized survivorship planning [64].

AI-enhanced QoL prediction models integrate clinical, genetic, and SDOH factors to estimate post-treatment functional outcomes, mental health effects, and overall well-being in cancer survivors. By leveraging real-world evidence (RWE) from EHRs, wearable health devices, and patient-reported outcome measures (PROMs), AI can help clinicians to make informed decisions regarding survivorship care plans and supportive interventions [65].

Future AI-driven cancer survivorship frameworks will likely incorporate reinforcement learning (RL) models that provide adaptive, patient-specific recommendations for follow-up care, dietary modifications, and psychological support. Predictive AI-based digital twins—virtual models that simulate individual patient responses—could revolutionize personalized post-treatment care strategies [66].

### 8.3. Emerging Trends in AI for Cancer SDOH Research

As AI becomes increasingly embedded in oncology decision making, the demand for transparency, accountability, and fairness in AI-driven predictions has led to the rise of explainable AI (XAI). Unlike traditional black-box deep learning models, XAI provides human-interpretable explanations for AI-generated cancer diagnoses, risk assessments, and treatment recommendations. Attention mechanisms, saliency maps, and Shapley Additive Explanations (SHAP) are among the emerging XAI techniques used to enhance trust in AI-driven oncology systems [67].

AI-driven community health interventions are also emerging as a pivotal strategy to reduce cancer disparities. By integrating SDOH variables into predictive models, AI can identify high-risk geographic regions and marginalized patient groups needing targeted cancer prevention initiatives. Future AI-powered digital health platforms will likely facilitate culturally tailored cancer education programs, mobile screening units, and AI-assisted telemedicine consultations for underserved populations [68].

Beyond treatment optimization, AI is increasingly deployed for cancer prevention strategies, incorporating environmental, behavioral, and socioeconomic risk factors. Traditional epidemiological studies have established that air pollution, occupational exposure to carcinogens, lifestyle habits (e.g., smoking, diet, physical activity), and socioeconomic barriers significantly contribute to cancer incidence. AI-powered environmental risk assessment models can now analyze satellite imagery, climate data, and pollution exposure metrics to predict geospatial cancer risk patterns [56].

## 9. Conclusions

Artificial intelligence (AI) is revolutionizing oncology by enhancing the diagnostic accuracy, optimizing treatment pathways, and improving survival outcomes, particularly for historically underserved populations. By integrating machine learning (ML), deep learning (DL), natural language processing (NLP), and predictive analytics, AI enables early cancer detection, personalized therapy selection, and data-driven clinical decision making. Incorporating social determinants of health (SDOH) into AI-driven oncology frameworks further strengthens efforts to reduce disparities in cancer screening, treatment access, and prognostic modeling. However, the ethical and equitable deployment of AI remains a critical challenge, with algorithmic bias, limited diversity in training datasets, and regulatory gaps posing significant barriers to fair AI implementation in cancer care.

To ensure that AI-driven oncology benefits all populations, bias mitigation strategies, fairness-aware machine learning models, and federated learning approaches must be prioritized. Developing explainable AI (XAI) frameworks will enhance their transparency, ensuring that AI-generated diagnoses and treatment recommendations are interpretable and clinically reliable. Furthermore, standardized regulatory frameworks must be developed to ensure data privacy, security, and ethical AI applications in oncology. Increasing the diversity in AI-driven clinical trials through AI-based recruitment strategies and decentralized learning will improve model generalizability and fairness in precision medicine.

For AI to truly bridge cancer care disparities, a multidisciplinary approach involving oncologists, AI researchers, policymakers, and bioethicists is essential. Future research should focus on producing diverse datasets, developing fairness-aware algorithms, and strengthening ethical oversight to prevent AI from perpetuating healthcare inequities. By prioritizing inclusivity, transparency, and patient-centered AI development, oncology can fully harness AI’s potential to advance precision medicine, equitable healthcare delivery, and improved cancer outcomes for all populations.

## Figures and Tables

**Table 1:** Social determinants of health (SDOH) factors and their impacts on cancer outcomes.

SDOH Factor	Impact on Cancer Outcomes	Most Associated Cancer Types
Socioeconomic Status (SES)	Low SES is linked to delayed diagnosis, reduced access to cancer screenings, financial constraints affecting treatment adherence, and lower participation in clinical trials.	Breast, Colorectal, Lung, Prostate
Healthcare Access and Insurance Coverage	Limited access to health insurance leads to fewer preventive screenings, higher out-of-pocket costs for cancer treatment, and disparities in the availability of advanced oncology care.	Breast, Cervical, Colorectal, Lung
Education Level	Lower education levels correlate with reduced awareness of cancer risk factors, decreased screening participation, and lower adherence to recommended treatments.	Colorectal, Cervical, Breast, Lung
Geographic Location (Urban vs. Rural)	Rural populations have reduced access to specialized oncology care, fewer early detection programs, and longer travel distances to treatment centers, leading to later-stage diagnoses.	Lung, Colorectal, Skin, Breast
Environmental Exposure	Higher exposure to air pollution, industrial chemicals, and carcinogens increases risks for lung, bladder, and liver cancers, particularly in low-income and minority communities.	Lung, Bladder, Liver, Mesothelioma
Housing Stability	Unstable housing conditions contribute to inconsistent healthcare access, missed oncology appointments, and increased exposure to environmental carcinogens.	Lung, Colorectal, Cervical
Employment and Occupational Hazards	Occupational exposure to carcinogens (e.g., asbestos, radiation, pesticides) is linked to higher incidences of lung, mesothelioma, and skin cancers, particularly among blue-collar workers.	Lung, Mesothelioma, Skin, Leukemia
Food Security and Nutrition	Poor nutrition and food insecurity lead to obesity-related cancers (e.g., colorectal, breast, pancreatic cancer) and deficiencies that impair immune function during cancer treatment.	Colorectal, Breast, Pancreatic, Liver
Psychosocial Stress and Mental Health	Chronic stress, depression, and a lack of social support negatively affect immune responses, treatment adherence, and overall survival outcomes in cancer patients.	Breast, Ovarian, Colorectal, Lung
Healthcare Literacy and Cultural Barriers	Language barriers, mistrust in medical institutions, and a lack of culturally competent healthcare limit participation in cancer prevention programs and impact treatment decisions.	Breast, Cervical, Prostate, Colorectal

**Table 2:** AI-driven interventions in different cancer types.

Cancer Type	AI-Powered Diagnostic Tools	AI-Driven Prognostic and Risk Assessment Models	AI-Based Treatment Optimization	Clinical Application and Benefits	Limitations
Breast Cancer	Deep learning-based mammography analysis, AI-driven ultrasound for dense breast tissue detection	Machine learning models predicting tumor recurrence and response to chemotherapy	AI-guided radiotherapy planning for dose precision, reinforcement learning models for chemotherapy regimen selection	Earlier detection in high-risk women, reduced false positives in mammography, and improved survival outcomes	Underperform in women with atypical tumor patterns due to biased training datasets
Lung Cancer	AI-enhanced CT scans for early lung nodule detection, deep learning-based PET imaging for metastasis evaluation	AI-based risk stratification for smokers and high-risk individuals, predictive modeling for immunotherapy response	Machine learning-based radiation therapy adaptation, AI-powered drug resistance prediction	Enhanced early-stage lung cancer detection, improved radiotherapy precision, and better immunotherapy patient selection	Risk of false positives, leading to unnecessary biopsies
Colorectal Cancer	AI-powered colonoscopy with real-time polyp detection, deep learning-based histopathology for tumor grading	Deep learning algorithms for prediction of colorectal cancer metastasis, AI-based survival prediction models	AI-driven robotic-assisted surgery for tumor resection, predictive analytics for personalized chemotherapy selection	Higher adenoma detection rates in colonoscopy, reduced colorectal cancer recurrence, and optimized chemotherapy regimens	High implementation costs; dependency on high-quality imaging and trained personnel
Prostate Cancer	MRI-based AI models for lesion classification, AI-driven biomarker detection in prostate-specific antigen (PSA) screening	AI-driven genomic profiling to stratify aggressive vs. indolent tumors, predictive modeling for active surveillance eligibility	AI-assisted focal therapy decision making, predictive modeling for hormone therapy responsiveness	Improved differentiation of aggressive vs. indolent tumors, enhanced radiotherapy targeting, and better patient selection for active surveillance	Limited interpretability of AI decisions; potential overtreatment or undertreatment due to model uncertainty
Liver Cancer	AI-powered liver elastography for fibrosis assessment, deep learning models for HCC detection in MRI and CT scans	AI-enhanced liver cirrhosis risk prediction, machine learning models for hepatic tumor recurrence	AI-driven radioembolization planning, predictive analytics for liver transplant success rates	Increased early detection rates for hepatocellular carcinoma (HCC), improved surgical planning, and better liver function preservation	Inconsistent performance in patients with comorbid liver diseases
Skin Cancer	AI-driven dermoscopy image classification for melanoma detection, convolutional neural networks (CNNs) for lesion differentiation	AI-assisted prognosis of melanoma progression, predictive modeling for immunotherapy outcomes	AI-enhanced image-guided surgery for melanoma excision, deep learning-based immunotherapy response prediction	More accurate melanoma classification, personalized immunotherapy strategies, and earlier intervention for high-risk patients	Risk of overfitting to fair-skinned datasets; limited accuracy in detecting atypical or rare skin lesions in dark skin tones
Brain Tumors	AI-assisted MRI segmentation for tumor localization, deep learning for glioblastoma grading	AI-powered survival prediction in glioblastoma patients, machine learning models for radiotherapy response assessment	AI-powered neurosurgical planning for precision tumor resection, AI-driven adaptive radiation therapy	Better tumor localization in surgical planning, optimized radiation dose for glioblastoma treatment, and improved long-term survival prediction	Limited data availability for rare brain tumors
Ovarian Cancer	AI-based ultrasound screening for early-stage ovarian tumors, deep learning models for histopathology-based tumor characterization	AI-driven ovarian cancer risk stratification based on genetic and lifestyle factors, predictive analytics for targeted therapies	AI-based monitoring of treatment response in ovarian cancer patients, AI-driven drug repurposing for personalized therapy	Improved early ovarian cancer detection, personalized therapeutic approaches, and enhanced chemotherapy effectiveness	Early-stage detection remains challenging due to vague symptoms; AI models require large-scale validation across populations

**Table 3:** AI applications in personalized cancer treatment.

AI Model Type	Precision Medicine Strategy	Clinical Applications	Key Benefits	Limitations
Deep Learning (DL) for Tumor Profiling	Analyzing histopathology, genomic, and imaging data to classify tumor subtypes and predict aggressiveness.	Predicting tumor behavior in breast, lung, and colorectal cancer; assisting pathologists in precision diagnosis.	Increases accuracy of tumor classification; enhances early detection and risk stratification.	Requires large labeled datasets; poor explainability.
Machine Learning (ML) for Drug Response Prediction	Based on molecular and clinical data to predict patient response to chemotherapy, targeted therapy, and immunotherapy.	Optimizing chemotherapy regimens for ovarian, pancreatic, and blood cancers; reducing adverse drug reactions.	Reduces treatment toxicity and improves survival rates through individualized drug selection.	Limited validation across minority populations.
AI-Powered Multi-Omics Integration	Integrating genomic, transcriptomic, proteomic, and metabolomic data to tailor individualized treatment plans.	Developing targeted liver, prostate, and brain cancer therapies; refining immunotherapy eligibility.	Provides a holistic view of disease biology, leading to more effective precision oncology interventions.	High computational cost and complexity; challenges in data harmonization across omics platforms.
Natural Language Processing (NLP) for Clinical Decision Support	Extracting key clinical insights from electronic health records (EHRs) and literature to recommend optimal treatments.	Supporting oncologists with real-time evidence-based treatment suggestions for rare and aggressive cancers.	Minimizes clinician workload; accelerates treatment planning for complex cancer cases.	Limited by variability in clinical note formats; risk of misinterpretation or loss of context in EHRs.
Reinforcement Learning (RL) for Adaptive Therapy	Developing dynamic, patient-specific treatment regimens that adapt based on tumor evolution and real-time response.	Personalizing radiation therapy plans for glioblastoma and prostate cancer; optimizing drug dosage in leukemia.	Enhances therapeutic outcomes by adjusting treatment in response to evolving cancer mutations.	Complex to train and validate; requires long-term real-time patient data.
Federated Learning for Global Precision Medicine	Training AI models across global healthcare institutions without data sharing, improving personalized therapy models.	Improving personalized treatment for rare cancers by leveraging multi-institutional datasets.	Maintains patient data privacy while enabling AI-driven global collaborations in oncology.	Technical challenges in model synchronization.
Explainable AI (XAI) for Treatment Transparency	Enhancing the interpretability of AI-driven treatment recommendations to improve clinician trust and patient adherence.	Providing transparent risk–benefit analysis of AI-generated treatment recommendations.	Increases trust in AI-driven decisions; facilitates regulatory approval of AI-based treatment recommendations.	Trade-off between model complexity and interpretability; XAI outputs are not clinically intuitive.
AI-Driven Biomarker Discovery	Identifying novel prognostic and predictive biomarkers for early cancer detection and treatment selection.	Advancing targeted drug discovery for triple-negative breast cancer, melanoma, and lung adenocarcinoma.	Enables discovery of next-generation biomarkers for early cancer detection and precision medicine.	Requires extensive validation and replication in diverse cohorts.

**Table 4:** AI data security techniques in cancer models.

Security Technique	Description	Use Case in AI-Driven Cancer Models	Key Benefits	Limitations/Challenges
Encryption (Homomorphic Encryption)	Ensures that data remain encrypted during AI model training and computation, preventing exposure of patient information.	Secure transmission of genomic data in AI-based precision oncology.	Prevents data leaks and ensures security in cloud-based AI training environments.	High computational cost and latency during encrypted operations; limited scalability for large datasets.
Anonymization and De-Identification	Removes personally identifiable information (PII) from datasets, allowing AI models to use data without compromising patient privacy.	Protecting patient identities in AI-powered cancer registries and clinical trials.	Enhances compliance with GDPR and HIPAA by minimizing data exposure risks.	Risk of re-identification through data triangulation.
Blockchain-Based Data Security	A decentralized security framework that provides tamper-proof records of AI-driven medical transactions and ensures data integrity.	Maintaining the integrity and security of AI-driven oncology decision-making systems.	It prevents data tampering and increases trust in AI-driven cancer diagnostics.	High energy consumption, scalability, and interoperability issues in large-scale healthcare systems.
Federated Learning	Enables AI model training across multiple institutions without sharing raw patient data, maintaining privacy while improving AI accuracy.	Collaborative AI training for multi-institutional cancer research while preserving patient confidentiality.	Allows AI models to be trained on diverse datasets without breaching patient confidentiality.	Requires complex model coordination; variations in local data quality may reduce model performance.
Differential Privacy	Adds statistical noise to datasets before AI processing, ensuring that individual data points cannot be re-identified while preserving trends.	Ensuring privacy in AI-driven predictive modeling for cancer risk assessment.	Balances privacy protection with AI-driven healthcare advancements.	Introduces accuracy trade-offs; fine-tuning the noise level is complex and context-dependent.
Access Control and Role-Based Authentication	Implements role-based access controls to restrict AI model interaction with sensitive patient data to authorized personnel only.	Restricting AI model access in hospitals and research centers to prevent unauthorized use.	Enhances cybersecurity and prevents unauthorized access to patient data in AI-driven systems.	Requires continuous monitoring and policy updates to stay secure.

**Table 5:** Regulatory frameworks for ethical AI implementation.

Regulatory Framework	Scope	Key Requirements	Impact on AI-Driven Cancer Models
HIPAA (Health Insurance Portability and Accountability Act, U.S.)	Regulates data privacy and security in AI-driven healthcare applications in the U.S.	Requires encryption, access control, and data anonymization in AI-driven cancer care.	Ensures patient data security in AI-powered oncology registries and diagnostic tools.
GDPR (General Data Protection Regulation, Europe)	Provides strict guidelines on AI data processing, patient consent, and data minimization in Europe.	Mandates explicit patient consent for AI data usage and allows individuals to request data deletion.	Protects patient rights in AI-driven cancer research and clinical trials.
FDA (U.S. Food and Drug Administration) AI/ML-Based Software Regulations	Ensures that AI-powered diagnostic and therapeutic models undergo validation, testing, and clinical safety evaluations.	Defines validation protocols for AI-driven imaging, pathology, and treatment recommendation systems.	Regulates the safety and efficacy of AI-powered cancer diagnostics and treatment systems.
Explainable AI (XAI) Guidelines	Mandates transparency in AI decision making, ensuring interpretability in AI-driven oncology applications.	Encourages the use of interpretable AI models in healthcare decision making.	Improves clinician trust and patient adoption of AI-driven personalized oncology treatments.
ISO/IEC 27001 (International Data Security Standard) [52]	Establishes standards for information security management in AI-driven medical research and clinical applications.	Requires AI-driven oncology models to follow strict data security and risk assessment protocols.	Enhances cybersecurity in AI-driven cancer data storage and processing platforms.

## Data Availability

All relevant data are contained within this manuscript. There are no additional datasets associated with this study.

## Abbreviations

The following abbreviations are used in this manuscript:

SDOH	Social Determinants of Health
AI	Artificial Intelligence
XAI	Explainable Artificial Intelligence
EHR	Electronic Health Record
NLP	Natural Language Processing
ML	Machine Learning
GIS	Geographic Information Systems
SES	Socioeconomic Status
HIPAA	Health Insurance Portability and Accountability Act
GDPR	General Data Protection Regulation
CNN	Convolutional Neural Network
RNN	Recurrent Neural Network
